# Phytotherapeutic Supplementation with *Momordica charantia*: Beneficial Effects in Patients with Suboptimal Glycemic Control on Double Antidiabetic Therapy—A Real-World Evidence Observational Study

**DOI:** 10.3390/nu18020309

**Published:** 2026-01-19

**Authors:** Cosmin Mihai Vesa, Timea Claudia Ghitea, Ada Radu, Andrei Flavius Radu, Teodora Maria Bodog, Ruxandra Florina Bodog, Roxana Daniela Brata, Cristiana Bustea

**Affiliations:** 1 Doctoral School of Biomedical Sciences, Faculty of Medicine and Pharmacy, University of Oradea, 410087 Oradea, Romania; adaradu@uoradea.ro (A.R.); andreiflavius.radu@uoradea.ro (A.F.R.); bodog.teodoramaria@student.uoradea.ro (T.M.B.); bodog.ruxandraflorina@student.uoradea.ro (R.F.B.); cbustea@uoradea.ro (C.B.); 2 Department of Preclinical Disciplines, Faculty of Medicine and Pharmacy, University of Oradea, 410073 Oradea, Romania; 3 Department of Pharmacy, Faculty of Medicine and Pharmacy, University of Oradea, 410028 Oradea, Romania; 4 Department of Psycho-Neuroscience and Recovery, Faculty of Medicine and Pharmacy, University of Oradea, 410073 Oradea, Romania; 5 Department of Surgical Disciplines, Faculty of Medicine and Pharmacy, University of Oradea, 410073 Oradea, Romania; 6 Department of Medical Disciplines, Faculty of Medicine and Pharmacy, University of Oradea, 410073 Oradea, Romania; brata.roxanadaniela@didactic.uoradea.ro

**Keywords:** type 2 diabetes, metformin, dapagliflozin, *Momordica charantia*, HbA1c, phytotherapy, glycemic control

## Abstract

Background: Phytotherapeutic agents, including *Momordica charantia*, have been proposed as complementary strategies to enhance metabolic control in type 2 diabetes mellitus patients on oral antidiabetic drugs. Methods: This was a real-world, longitudinal, comparative cohort study with treatment escalation, conducted in patients with type 2 diabetes mellitus receiving metformin therapy. All patients were initially prescribed add-on dapagliflozin at 10 mg/day and re-evaluated after 6 months. Based on glycemic response at 6 months, patients were stratified into two groups: 70 patients with persistent inadequate glycemic control received adjunctive supplementation with a standardized *Momordica charantia* extract for 3 months (intervention group), while 85 patients who achieved glycemic targets continued dual antidiabetic therapy alone (control group). Anthropometric, hemodynamic, and metabolic parameters were assessed at baseline, 6 months, and 9 months from baseline. Results: Between-group analyses revealed divergent glycemic trajectories during the 6–9 month interval. In the intervention group, HbA1c decreased from 7.82 ± 0.58% at baseline to 6.93 ± 0.30% at 6 months and to 6.34 ± 0.42% at 9 months, while in the control group, glycemic parameters showed only modest additional changes after 6 months. The reduction in HbA1c and fasting plasma glucose between 6 and 9 months was significantly greater in patients receiving *Momordica charantia* compared with controls (*p* < 0.001). Fasting plasma glucose declined from 138.4 ± 17.5 mg/dL at baseline to 122.3 ± 13.1 mg/dL at 6 months and to 113.3 ± 12.2 mg/dL at 9 months in the intervention group. Dapagliflozin therapy was associated with significant improvements in body weight, BMI, and blood pressure at 6 months in both groups, whereas adjunctive *Momordica charantia* supplementation did not produce significant additional effects on anthropometric or hemodynamic parameters. Conclusions: Adjunctive *Momordica charantia* supplementation was associated with additional improvements in glycemic control compared with continuation of dual antidiabetic therapy alone, with the most pronounced effects observed for HbA1c and fasting plasma glucose. These findings support a potential adjunctive role for phytotherapeutic supplementation in patients with suboptimal glycemic control receiving contemporary standard therapy.

## 1. Introduction

Type 2 diabetes mellitus (T2DM) is a major worldwide health burden, defined by chronic hyperglycemia and linked to long-term consequences such as cardiovascular disease, nephropathy, neuropathy, and retinopathy [1]. Despite advancements in pharmaceutical therapy such as sodium glucose cotransporter 2 (SGLT2) inhibitors and glucagon-like peptide 1 (GLP-1) agonists, a significant minority of patients fail to achieve and maintain adequate glycemic control, emphasizing the need for innovative and integrative treatment strategies such as plant supplements or plant extracts [1,2,3]. Phytochemicals have been well known to have therapeutical and even healing effects since thousands of years ago [4], in addressing the numerous diseases and pathophysiological pathways such as increased oxidative stress, insulin resistance, pro-inflammatory pathways, etc. [3].

Metformin remains the cornerstone of first-line pharmacotherapy due to its efficacy, safety, and cardiovascular benefits, as reflected in long fallow-up studies such as UKPDS [5,6]. For patients with insufficient control on metformin monotherapy, SGLT2 inhibitors such as dapagliflozin are widely used and have demonstrated improvements in both metabolic and cardiovascular outcomes having numerous pleiotropic effects such as reducing heart failure aggravation risk or protecting the renal function of T2DM patients [7,8]. Nevertheless, many patients continue to exhibit suboptimal glycemic control, motivating interest in complementary approaches to enhance standard therapy. It is also important to mention the numerous side effects of antidiabetic medication including urinary tract infections, dehydration, euglycemic ketoacidosis for SGLT2-inhibitors or pancreatitis, gallbladder function aggravation for GLP-1 agonists [9].

A systematic review found that natural products such as bitter melon, cinnamon or whortleberry supplements, *Berberis aristata*/*Silybum marianum*, fenugreek seed, *Nigella sativa*, Mulberry juice, chicory, chamomile tea, and bell pepper juice, combined with an integrated approach of yoga therapy, were effective for glycemic control in T2DM [10,11]. Plant extracts often inhibit α-glucosidase and advanced glycation end products (AGE) production, enhance glucose transporter type 4 (GLUT-4) and peroxisome proliferator-activated receptors (PPARs) expression, and provide antioxidant properties [9,10]. Among these, *Momordica charantia* (bitter melon) has been traditionally used in various cultures for its antidiabetic properties. *Momordica charantia*’s phytochemical composition includes a wide range of bioactive constituents, such as triterpenoids, saponins, alkaloids, flavonoids, phenolic compounds, and polypeptide-p (a plant insulin analog), all of which contribute to its antidiabetic efficacy via multiple biochemical pathways. Furthermore, substances such as charantin, vicine, and momordicin have synergistic antioxidant and lipid-lowering actions, which reduce oxidative stress and improve insulin sensitivity [12,13]. *Momordica charantia* has hypoglycemic and metabolic-modulating effects through a variety of pathways, including increased insulin sensitivity, stimulation of glucose uptake, inhibition of hepatic gluconeogenesis, and control of lipid metabolism [13]. Triterpenoid compounds, including charantin and momordicins, have been shown in experimental models to modulate key nodes of insulin signaling, particularly through activation of AMP-activated protein kinase (AMPK) and downstream enhancement of glucose transporter type 4 (GLUT-4) translocation in skeletal muscle and adipose tissue, thereby facilitating peripheral glucose uptake independently of insulin availability [12]. Saponins, which frequently coexist with triterpenoids in bitter melon extracts, appear to exert complementary effects by inhibiting intestinal α-glucosidase and α-amylase activity, resulting in delayed carbohydrate absorption and attenuation of postprandial hyperglycemia, while also exhibiting anti-inflammatory and antioxidant properties that may indirectly improve insulin receptor signaling by reducing oxidative stress–induced insulin resistance [13]. In contrast, polypeptide-p, a plant-derived insulin-like peptide, has been reported to exert insulin-mimetic effects in preclinical studies, promoting glucose uptake and glycogen synthesis; however, its clinical relevance remains uncertain due to limited data on oral bioavailability, stability, and systemic absorption in humans [12,13]. Despite increasing interest in as a potential adjunct in the management of type 2 diabetes mellitus, the available clinical evidence remains inconsistent and difficult to interpret. Many previously published studies are characterized by substantial heterogeneity in extract formulation, phytochemical composition, dosing strategies, and duration of intervention, limiting comparability and reproducibility of results [9,10]. In addition, several trials have enrolled poorly characterized or mixed patient populations, often without detailed reporting of baseline metabolic control, disease duration, comorbidity burden, or background antidiabetic therapy [10,11]. Importantly, most clinical investigations evaluating *Momordica charantia* were conducted prior to the widespread adoption of contemporary glucose-lowering agents and there is a notable lack of data assessing its use in patients treated with SGLT2 inhibitors, despite their central role in current diabetes management. Collectively, these methodological and contextual gaps have hindered definitive conclusions regarding the clinical utility of and underscore the need for observational data in well-defined populations receiving modern standard-of-care therapy.

In this context, the present study aimed to assess the effect of *Momordica charantia* supplementation on anthropometric, hemodynamic, and metabolic parameters in patients with T2DM treated with modern antidiabetic therapy that did not achieving glycemic control. We hypothesized that *Momordica charantia* supplementation would provide additional glycemic and metabolic benefits beyond conventional therapy, particularly reflected in reductions in HbA1c and fasting glucose.

## 2. Materials and Methods

### 2.1. Study Design and Participants

This study included patients with type 2 diabetes mellitus (T2DM), followed in SC Elias Invest Medimages SRL clinic, Oradea, Romania, aiming to evaluate the impact of adjunctive phytotherapeutic supplementation on glycemic and cardiometabolic control in patients receiving double antidiabetic therapy. This investigation represents a real-world, observational cohort study with a comparative design, reflecting routine clinical decision-making and treatment escalation in patients with T2DM receiving contemporary standard-of-care therapy. As such, the study was designed to analyze longitudinal glycemic trajectories and between-group differences in a real-life clinical setting rather than to evaluate the efficacy of an intervention under controlled trial conditions. Eligible participants were adults aged 18–75 years, previously diagnosed with T2DM according to ADA criteria, with inadequate glycemic control on metformin monotherapy (HbA1c ≥ 6.5%). Patients with severe hepatic or renal impairment, active malignancy, or those receiving insulin therapy were excluded. All participants provided informed consent prior to inclusion. The study was conducted between March 2024 to September 2025.

This study was conducted in accordance with the Declaration of Helsinki and was approved by the Institutional Review Board of the Faculty of Medicine and Pharmacy, University of Oradea (approval no. 5/30 October 2023).

### 2.2. Intervention Protocol

Initially, 155 consecutive patients treated with metformin monotherapy and presenting inadequate glycemic control (HbA1c ≥ 6.5%) underwent baseline evaluation (T0). All were prescribed an additional antidiabetic agent, dapagliflozin 10 mg/day, selected for its proven cardio-renal benefits and low risk of hypoglycemia.

After 6 months of combination therapy with metformin and dapagliflozin (T1), patients were stratified according to their glycemic response. Seventy patients (45.2%) who continued to exhibit inadequate glycemic control (HbA1c ≥ 6.5%) were allocated to the intervention group, while eighty-five patients who achieved glycemic control (HbA1c < 6.5%) constituted the control group.

At T1, patients in the intervention group initiated supplementation with a standardized *Momordica charantia* extract (3% bitter compounds), administered orally at a dose of 450 mg three times daily after meals, in addition to ongoing metformin and dapagliflozin therapy. Patients in the control group continued treatment with metformin and dapagliflozin without phytotherapeutic supplementation.

The third evaluation (T2) was performed after 3 months (i.e., 9 months from baseline). Both the intervention and control groups underwent identical clinical and laboratory evaluations at all study time points (T0, T1, and T2). Concomitant medications for hypertension or dyslipidemia were permitted provided that doses remained stable throughout the study period.

### 2.3. Clinical and Laboratory Assessments

The study involved three evaluations, defined as follows:

- Baseline evaluation (T0)—Patients on metformin monotherapy not achieving optimal glycemic control. The decision to add dapagliflozin was made at this moment.
- 6 months evaluation (T1)—After 6 months of add-one therapy with dapagliflozin 10 mg/day.
- 9 months evaluation from baseline (T2)—After 3 months of *Momordica charantia* supplementation.

The following parameters were recorded:

- Anthropometric: body mass index (BMI), body weight (kg).
- Hemodynamic: systolic blood pressure (SBP), diastolic blood pressure (DBP).
- Metabolic: HbA1c (%), fasting blood glucose (mg/dL).

All measurements were performed under standardized conditions: weight and BMI were calculated using calibrated scales, blood pressure was measured in the seated position after 5 min of rest, blood glucose was determined using glucose oxidase method after an overnight fast and HbA1c was measured from venous samples using HPLC technique.

### 2.4. Statistical Analysis

Continuous variables were expressed as mean ± standard deviation (SD), and categorical variables as counts and percentages. All analyses included participants with complete data across all three time points (T0, T1, and T2; *n* = 155).

The primary endpoint was the between-group difference in HbA1c change from T1 to T2 (ΔHbA1c T1–T2). Longitudinal comparisons between baseline (T0), the 6-month dapagliflozin evaluation (T1), and the 9-month *Momordica charantia* supplementation phase (T2) were performed exclusively in participants with complete data for all three time points (*n* = 155). Between-group comparisons of continuous outcomes were performed using independent-samples *t*-tests or analysis of covariance (ANCOVA), as appropriate. ANCOVA models were adjusted for age, sex, baseline HbA1c (T0), and HbA1c at T1 to account for baseline differences between groups.

For continuous outcomes (BMI, body weight, systolic/diastolic blood pressure, fasting plasma glucose, and HbA1c), paired-samples *t*-tests were used to evaluate changes between T0–T1, T1–T2, and T0–T2. Normality of paired differences was assessed using the Shapiro–Wilk test. When non-normality was detected, Wilcoxon signed-rank tests were additionally inspected to confirm robustness. To account for repeated paired comparisons, *p*-values were adjusted using the Bonferroni correction (α_adj_ = 0.05/3 = 0.0167) or the Holm method where indicated. Effect sizes were calculated using Cohen’s dz, with values ≥ 0.80 interpreted as large effects. Effect sizes reflect magnitude of within-group change and should not be interpreted as evidence of causal efficacy.

Changes in the proportion of participants achieving the ADA fasting glucose target (<130 mg/dL) between 6 and 9 months were analyzed using McNemar’s test for paired categorical outcomes.

To explore determinants of glycemic response, multiple linear regression models were constructed with the absolute reduction in HbA1c (T0–T2) as the dependent variable. Independent variables included clinically relevant comorbidities (hypertension, heart failure, chronic kidney disease, diabetic retinopathy, peripheral neuropathy), age, sex, and baseline HbA1c. Model assumptions (linearity, homoscedasticity, normality of residuals, multicollinearity) were verified using standardized residual plots, Durbin–Watson statistics, and variance inflation factors (VIF).

All statistical tests were two-tailed, and statistical significance was defined as *p* < 0.05 unless adjusted otherwise. Analyses were performed using IBM SPSS Statistics, Version 30 (IBM Corp., Armonk, NY, USA).

### 2.5. Ethical Approval

Clinical trial registration was not required, as this study was observational and involved approved antidiabetic medications (metformin and dapagliflozin) used according to national and international guidelines, including those of the American Diabetes Association. The initiation of dapagliflozin represented a standard clinical decision for patients not achieving glycemic targets on metformin monotherapy. *Momordica charantia* supplementation is not regulated as an investigational medicinal product in Romania and does not fall under the national clinical trial regulatory framework.

## 3. Results

### 3.1. Baseline Characteristics of the Study Population

The study cohort consisted of 155 patients with type 2 diabetes mellitus, who were stratified at the 6-month evaluation (T1) into an intervention group (*n* = 70) and a control group (*n* = 85) based on their glycemic response to dual antidiabetic therapy.

At baseline (T0), the mean age of the entire cohort was comparable between groups, with a mean age of 61 ± 9 years in the intervention group and 60 ± 8 years in the control group. Women represented 54.3% (*n* = 38) of the intervention group and 52.9% (*n* = 45) of the control group, while men accounted for the remaining participants, with no statistically significant difference in sex distribution between groups.

The duration of diabetes was heterogeneously distributed in both groups, with no significant between-group differences. In the intervention group, 17.1% had a disease duration of 1 year, 24.3% of 2 years, 12.9% of 3 years, 20.0% of 4 years, 14.3% of 5 years, 4.3% of 6 years, and 7.1% of 8 years. A similar distribution was observed in the control group, indicating comparable disease chronicity at baseline.

Regarding microvascular complications, peripheral neuropathy was present in 54.3% of patients in the intervention group and 49.4% in the control group, while diabetic retinopathy was identified in 20.0% and 18.8% of patients, respectively. Chronic kidney disease was documented in 18.6% of the intervention group and 16.5% of the control group.

As for macrovascular comorbidities, hypertension was present in 51.4% of patients receiving Momordica supplementation and in 49.4% of control patients, while congestive heart failure was identified in 18.6% and 17.6% of patients, respectively. No statistically significant differences in baseline demographic or clinical characteristics were observed between the two groups, supporting their suitability for comparative longitudinal analysis.

The baseline demographic and clinical characteristics of both groups are summarized in Table 1.

### 3.2. Parameter Evolution and Metabolic Outcomes

At baseline (T0), all enrolled patients (*n* = 155) were receiving metformin monotherapy. Baseline anthropometric, hemodynamic, and metabolic parameters were comparable between the intervention and control groups, as detailed in Table 1.

In the intervention group (*n* = 70), the mean BMI at baseline was 30.57 ± 2.21 kg/m^2^, with an average body weight of 95.9 ± 14.1 kg. Baseline blood pressure values were 140.7 ± 12.6 mmHg (systolic) and 97.4 ± 15.9 mmHg (diastolic). Mean HbA1c was 7.82 ± 0.58%, and fasting plasma glucose was 138.4 ± 17.5 mg/dL.

In the control group (*n* = 85), baseline values showed a similar cardiometabolic profile, confirming the absence of significant between-group differences at study entry.

After 6 months of dapagliflozin add-on therapy (T1), both groups exhibited significant improvements in glycemic and cardiometabolic parameters, reflecting the known metabolic effects of SGLT2 inhibition. In the intervention group, BMI decreased to 29.56 ± 2.33 kg/m^2^, body weight to 92.8 ± 13.9 kg, systolic blood pressure to 133.5 ± 11.6 mmHg, and diastolic blood pressure to 93.5 ± 14.6 mmHg. Mean HbA1c declined to 6.93 ± 0.30%, and fasting plasma glucose to 122.3 ± 13.1 mg/dL.

At this time point (T1), all patients allocated to the intervention group continued to exhibit inadequate glycemic control (HbA1c ≥ 6.5%), which represented the criterion for the initiation of *Momordica charantia* supplementation.

Between T1 and T2, divergent glycemic trajectories were observed between the two study groups.

In the intervention group, following 3 months of adjunctive *Momordica charantia* supplementation, further metabolic improvements were recorded. BMI decreased slightly to 29.42 ± 2.41 kg/m^2^, while body weight remained relatively stable (92.5 ± 13.9 kg). Blood pressure values showed minimal additional change (133.0 ± 9.7/93.3 ± 13.5 mmHg). Notably, HbA1c decreased to 6.34 ± 0.42%, and fasting plasma glucose improved to 113.3 ± 12.2 mg/dL.

In contrast, patients in the control group, who continued metformin and dapagliflozin without phytotherapeutic supplementation, demonstrated only modest additional changes in glycemic parameters between T1 and T2, with stabilization rather than marked further reduction in HbA1c and fasting plasma glucose.

Overall, these findings indicate that while anthropometric and blood pressure changes were largely was associated with dapagliflozin therapy and plateaued during follow-up, adjunctive *Momordica charantia* supplementation was associated with a more pronounced improvement in glycemic control between T1 and T2 compared with continuation of dual antidiabetic therapy alone. Detailed longitudinal data for the intervention group are presented in Table 2, while between-group comparisons are addressed in subsequent analyses.

**Figure 1:**
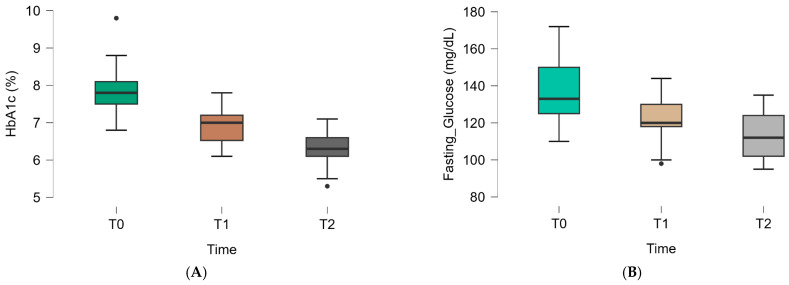
Evolution of HbA1c (**A**) and fasting plasma glucose (**B**) across treatment phases: baseline (T0, metformin monotherapy), 6 months (T1, dapagliflozin add-on), and 9 months (T2, *Momordica charantia* supplementation). Data are shown as mean ± SD (*n* = 155). Dots represent outliers, defined as individual observations that fall outside the whisker range of the boxplot.

Progressive improvements in glycemic control were observed across treatment phases in the intervention group. Compared with baseline values, dapagliflozin addition was associated with a marked reduction in both HbA1c and fasting plasma glucose. Following the introduction of *Momordica charantia*, a further decline in glycemic parameters was observed, exceeding that seen in the control group during the same interval, as illustrated in Figure 1.

### 3.3. Paired Comparison of Anthropometric, Blood Pressure, and Glycemic Parameters

Within-group longitudinal analyses were performed exclusively in the intervention group (*n* = 70) to characterize temporal changes across the three evaluation points and to contextualize the metabolic response observed following treatment escalation.

Both HbA1c and fasting plasma glucose demonstrated a progressive and statistically significant reduction from baseline (T0) to 6 months (T1) and from T1 to 9 months (T2) in patients receiving adjunctive *Momordica charantia* supplementation.

HbA1c decreased from 7.82 ± 0.58% at baseline (T0) to 6.93 ± 0.30% after six months of dapagliflozin therapy (T1) and further to 6.34 ± 0.42% at nine months (T2). Paired-samples *t*-tests confirmed significant differences between all time points:

- T0–T1: Δ = −0.89% (95% CI: −1.05 to −0.73), *p* < 0.001, Cohen’s dz = 1.25;
- T1–T2: Δ = −0.59% (95% CI: −0.72 to −0.46), *p* < 0.001, Cohen’s dz = 0.98;
- T0–T2: Δ = −1.48% (95% CI: −1.66 to −1.30), *p* < 0.001, Cohen’s dz = 1.75.

All *p*-values remained statistically significant following Bonferroni correction (α_adj = 0.0167).

A similar pattern was observed for fasting plasma glucose, which declined from 138.4 ± 17.5 mg/dL (T0) to 122.3 ± 13.1 mg/dL (T1) and 113.3 ± 12.2 mg/dL (T2). Mean paired differences were as follows:

- T0–T1: Δ = −16.1 mg/dL (95% CI: −20.9 to −11.3), *p* < 0.001, Cohen’s dz = 1.09;
- T1–T2: Δ = −9.0 mg/dL (95% CI: −12.7 to −5.3), *p* < 0.001, Cohen’s dz = 0.83;
- T0–T2: Δ = −25.1 mg/dL (95% CI: −29.8 to −20.4), *p* < 0.001, Cohen’s dz = 1.56.

**Figure 2:**
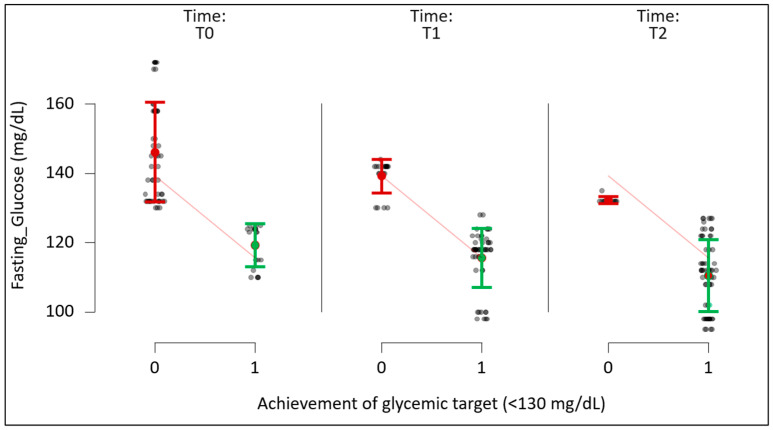
Distribution of fasting plasma glucose according to achievement of the glycemic target (<130 mg/dL) across study time points in the intervention and control groups: T0—baseline; T1—6 months after dual antidiabetic therapy; T2—after 3 months of *Momordica charantia* supplementation. Black dots represent individual participant fasting glucose values. Red and green dots indicate group means for participants not achieving (0, red) and achieving (1, green) the glycemic target (<130 mg/dL), respectively. Error bars denote standard deviation. Sloped red lines illustrate the mean difference in fasting glucose between groups at each time point.

Normality of paired differences was confirmed using the Shapiro–Wilk test (all *p* > 0.05). The magnitude and consistency of glycemic improvement, together with large within-group effect sizes (Cohen’s dz ≥ 0.8), indicate a clinically relevant reduction in glycemic burden following sequential therapeutic escalation. Corresponding trajectories are illustrated in Figure 2, and detailed statistics are summarized in Table 3.

In contrast, anthropometric and blood pressure parameters exhibited a biphasic response pattern. During the first 6 months (T0–T1), BMI and body weight decreased significantly by −1.01 kg/m^2^ and −3.01 kg, respectively (*p* < 0.001), accompanied by reductions in systolic (−7.19 mmHg) and diastolic blood pressure (−3.90 mmHg). These changes are consistent with the known cardiometabolic effects of SGLT2 inhibition.

Between T1 and T2, corresponding to the *Momordica charantia* supplementation phase, anthropometric and hemodynamic parameters remained largely stable (all *p* > 0.05), indicating a plateau effect rather than further reduction. Nevertheless, HbA1c and fasting plasma glucose continued to decline significantly, suggesting that the additional glycemic benefit observed during this phase was not mediated by further weight loss or blood pressure changes, but rather reflects a complementary glucose-lowering mechanism.

Overall, the paired analyses presented in Table 3 support a two-phase metabolic response in the intervention group: an initial cardiometabolic improvement driven predominantly by dapagliflozin therapy, followed by a sustained and independent glycemic benefit during adjunctive *Momordica charantia* supplementation.

### 3.4. Glycemic Target Achievement and Population Distribution

To further characterize the metabolic response to therapy, we evaluated the proportion of patients achieving a clinically relevant fasting plasma glucose target (<130 mg/dL) over time and compared target attainment between study groups.

At baseline (T0), 28.6% of patients in the intervention group (*n* = 20) and 30.6% of patients in the control group (*n* = 26) achieved the fasting glucose target. After 6 months of dual therapy with metformin and dapagliflozin (T1), the proportion of patients reaching the target increased substantially in both groups, to 71.4% (*n* = 50) in the intervention group and 74.1% (*n* = 63) in the control group, reflecting the robust glucose-lowering effect of SGLT2 inhibition.

Between T1 and T2, divergent patterns of glycemic target attainment were observed. Following 3 months of adjunctive *Momordica charantia* supplementation, the proportion of patients in the intervention group with fasting plasma glucose < 130 mg/dL increased further to 87.1% (*n* = 61). In contrast, only a modest additional increase was observed in the control group, in which 78.8% (*n* = 67) of patients achieved the target at T2.

Within-group analyses confirmed statistically significant improvements in target attainment over time in the intervention group (T0 vs. T1 and T0 vs. T2, both *p* < 0.01), whereas the incremental change between T1 and T2 was significantly greater in the intervention group compared with the control group (*p* < 0.05).

Consistent with these categorical findings, the mean reduction in fasting plasma glucose from T1 to T2 was −9.0 mg/dL in the intervention group, compared with −2.1 mg/dL in the control group, indicating a greater downward shift in glycemic distribution among patients receiving phytotherapeutic supplementation.

Collectively, these results demonstrate that while dual antidiabetic therapy markedly increased the proportion of patients achieving near-normoglycemia, adjunctive *Momordica charantia* supplementation was associated with a higher likelihood of attaining and maintaining fasting glucose targets, supporting its role as an effective adjunctive strategy in the multimodal management of type 2 diabetes.

### 3.5. Predictors of Glycemic Response: Multiple Linear Regression Analysis

To identify clinical and treatment-related predictors of glycemic improvement, multivariable linear regression analyses were performed using the change in HbA1c between T1 and T2 (ΔHbA1c T1–T2) as the dependent variable, corresponding to the period of adjunctive *Momordica charantia* supplementation or continuation of dual therapy (Table 4).

All regression models included study group (intervention vs. control) as a key independent variable, together with relevant demographic and clinical covariates. Covariates were selected a priori based on clinical relevance and included age, sex, baseline HbA1c (T0), HbA1c at T1, duration of diabetes, and major diabetes-related comorbidities (hypertension, heart failure, chronic kidney disease, diabetic retinopathy, and peripheral neuropathy).

In unadjusted models, membership in the intervention group was significantly associated with a greater reduction in HbA1c between T1 and T2 (β ≈ −0.38, *p* < 0.001), confirming the between-group findings observed in the primary comparative analyses.

After adjustment for demographic variables and comorbidities, the study group remained the strongest independent predictor of HbA1c reduction, while none of the evaluated comorbidities significantly modified the glycemic response. Heart failure showed a borderline positive association with HbA1c reduction (β ≈ 0.26, *p* ≈ 0.05), whereas hypertension demonstrated a non-significant inverse trend (β ≈ −0.24, *p* ≈ 0.07). Chronic kidney disease, diabetic retinopathy, peripheral neuropathy, and disease duration were not significantly associated with ΔHbA1c (all *p* > 0.1).

**Figure 3:**
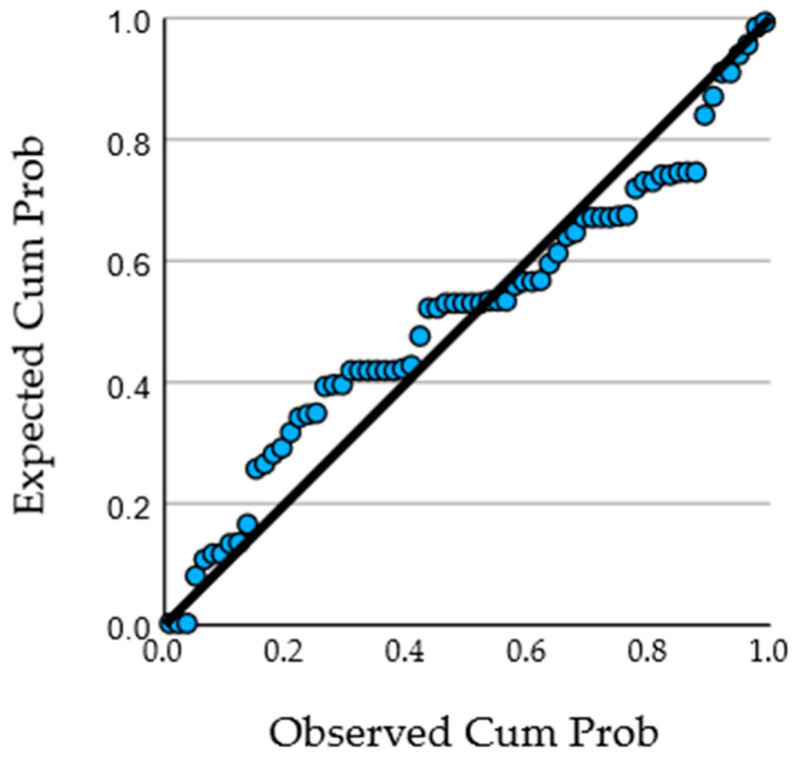
Normal P–P plot of standardized residuals for the multivariable regression model using ΔHbA1c (T1–T2) as the dependent variable, demonstrating acceptable model assumptions.

Overall model fit was modest, with ANOVA *p*-values > 0.05 in fully adjusted models, indicating that baseline clinical characteristics explained only a limited proportion of interindividual variability in glycemic response beyond treatment allocation. Residual diagnostics confirmed normal distribution, homoscedasticity, and absence of influential outliers, supporting the validity of the regression assumptions (Figure 3).

Collectively, these findings suggest that the observed improvement in HbA1c during the T1–T2 interval was observed during the Momordica supplementation phase, rather than by baseline demographic or comorbidity profiles, indicating that a relatively weak effect of modification was detected across patient subgroups.

### 3.6. Summary of Key Findings and Robustness of Results

Taken together, the results of this real-world comparative cohort study indicate a two-stage metabolic response in patients with type 2 diabetes undergoing treatment escalation. The initial introduction of dapagliflozin on a background of metformin therapy (T0–T1) was associated with substantial improvements in glycemic control, anthropometric parameters, and blood pressure in both study groups, reflecting the established cardiometabolic benefits of SGLT2 inhibition.

During the subsequent follow-up period (T1–T2), distinct glycemic trajectories emerged between groups. Patients receiving adjunctive *Momordica charantia* supplementation demonstrated a significantly greater reduction in HbA1c and fasting plasma glucose compared with patients continuing dual antidiabetic therapy alone. This between-group difference was consistently observed across multiple analytical approaches, including absolute change analyses, categorical glycemic target attainment, and multivariable regression models adjusted for baseline glycemic status and clinical covariates.

Importantly, the additional glycemic improvement observed in the intervention group occurred in the absence of further reductions in body weight or blood pressure, suggesting that the effect of *Momordica charantia* was not mediated by hemodynamic or anthropometric changes but rather reflected a complementary glucose-lowering mechanism. This interpretation is further supported by the persistence of the group effect after adjustment for age, sex, baseline HbA1c, diabetes duration, and major comorbidities.

Sensitivity analyses confirmed the robustness of the findings, with consistent directionality and magnitude of glycemic changes across unadjusted and adjusted models. No major effect modification by baseline comorbidities was detected, although limited power cannot be excluded.

Overall, these results support the potential role of *Momordica charantia* as an effective adjunct to contemporary dual antidiabetic therapy in patients with suboptimal glycemic control, within the context of routine clinical practice.

## 4. Discussion

This study investigated the impact of treatment escalation—from metformin monotherapy to the addition of dapagliflozin 10 mg/day and subsequent Momordica supplementation—on cardiometabolic parameters in patients with type 2 diabetes. Using a real-world comparative cohort design, our findings indicate that while modest improvements were observed in BMI, body weight, and blood pressure after *Momordica charantia* supplementation, glycemic control—as evaluated by fasting plasma glucose and HbA1c—improved significantly and to a greater extent compared with continuation of dual antidiabetic therapy alone.

These results are consistent with previous reports highlighting the incremental benefits of combining pharmacological therapy with phytotherapeutic agents to achieve optimal glycemic targets [14,15]. Our findings suggest that *Momordica charantia* may be associated with additional glucose-lowering effects beyond contemporary standard pharmacological therapy. Potential mechanisms include modulation of insulin sensitivity, inhibition of intestinal glucose absorption, and enhancement of pancreatic β-cell function, as described in experimental studies [16,17,18,19,20]. These mechanisms remain speculative in the context of the present study. While numerous studies support the beneficial effect of *Momordica charantia* on glycemic control, there are also findings reporting no beneficial effects compared to placebo [18]. *Momordica charantia* is among other plant supplements such as *Gymnema sylvestre*, *Stachytarpheta jamaicensis*, *Physalis angulata* L., *Stachytarpheta jamaicensis* commonly used for improving glycemic control in individuals on traditional antidiabetic therapy data from literature demonstrating that α-glucosidase activity inhibition is one of the main sites on action of these plant supplements [19,20].

In contrast, changes in BMI and blood pressure, although statistically significant in some comparisons, were modest in magnitude. Notably, between-group analyses demonstrated that additional glycemic improvements occurred in the absence of further reductions in body weight or blood pressure, suggesting that the primary benefit of Momordica charantia supplementation was metabolic rather than hemodynamic. This observation suggests that the primary benefit of the intervention was metabolic rather than hemodynamic, aligning with evidence that short-term adjunctive therapies may exert a stronger impact on glucose metabolism than on weight or vascular parameters [21,22,23,24].

Our regression analysis further showed that common comorbidities such as hypertension, heart failure, and chronic kidney disease did not independently predict HbA1c variation after adjustment for treatment group. This highlights the potential of therapeutic optimization—including novel adjuncts such as Momordica—to achieve glycemic improvements regardless of comorbidity burden [25,26].

Taken together, these findings support the role of integrated pharmacological and phytotherapeutic strategies in enhancing glycemic control in patients with type 2 diabetes. However, given the observational nature of the study and the non-randomized allocation of treatment escalation, causal inference should be interpreted with caution. Larger, controlled trials are warranted to confirm these observations, explore long-term outcomes, and better elucidate the mechanisms underlying the observed effects.

Beyond the observed glycemic benefits, our findings also align with emerging evidence suggesting that phytotherapeutic agents such as *Momordica charantia* may complement established pharmacological regimens in a multifaceted way. Recent systematic reviews and meta-analyses indicate that while the magnitude of glucose-lowering effects varies, the overall trend supports clinically meaningful reductions in HbA1c when *Momordica charantia* is used as an adjunct to standard care [18,27]. This resonates with our results, which demonstrate incremental glycemic improvements despite patients already receiving metformin and an SGLT2 inhibitor.

Another relevant aspect is the potential pleiotropic benefits of Momordica. Experimental and translational studies suggest anti-inflammatory, antioxidant, and lipid-modulating properties that may indirectly contribute to improved metabolic health [25,28,29]. These effects are of particular interest given that chronic low-grade inflammation and oxidative stress are major drivers of diabetic complications [30]. Although our study did not directly assess these pathways, the persistence of glycemic benefits after adjustment for baseline clinical characteristics supports the hypothesis of a direct metabolic effect.

It is also worth noting that the modest effects on body weight and blood pressure observed in our study are consistent with reports indicating that Momordica’s primary influence is on glucose metabolism rather than hemodynamic regulation [31,32]. However, in contrary with our findings some studies have reported beneficial effects on lipid metabolism and cardiovascular markers, suggesting possible longer-term cardioprotective benefits that warrant further investigation [33,34].

Momordicine I, derived from *Momordica charantia*, has demonstrated potential blood pressure-lowering effects. The proposed mechanisms include inhibition of the renin–angiotensin system and increased nitric oxide production [35]. While some studies support these findings, others have reported no significant effect. On one hand, the review by Kao et al. [25] highlighted the beneficial cardiovascular actions of *Momordica charantia*, which were attributed not only to direct RAS inhibition and nitric oxide modulation but also to its hypoglycemic, lipid-lowering, and anti-inflammatory properties. On the other hand, Eszter Laczkó-Zöld et al. [18], in their systematic review and meta-analysis of randomized clinical trials, concluded that the metabolic effects of *Momordica charantia* could not be clearly determined based on the available clinical evidence. In their analysis, the extract showed no significant impact on blood pressure. However, a key limitation of the analyzed studies was the short follow-up duration, typically between 4 and 12 weeks. Given that *Momordica charantia* influences several components of metabolic syndrome, its effects on blood pressure may become evident only after a longer period. In our study, the 3-month supplementation period may have been insufficient to detect measurable blood pressure effects, potentially explaining the neutral hemodynamic findings.

The fact that regression analysis demonstrated that comorbidities did not significantly affect the glycemic response to Momordica may reflect the broad metabolic activity of the supplement, which seems to retain efficacy even in patients with complex clinical profiles. Such results are encouraging for clinical translation, as real-world patients often present with multiple comorbidities [27,36].

An emerging area of interest is the use of plant-based combinations for diabetes management. T2DM patients could benefit not only from one plant supplement administration as adjunct to their therapy but plant combinations could potentially possess better clinical effects. Several clinical and preclinical studies have shown that combinations of bitter melon with other botanicals such as *Gymnema sylvestre*, *Trigonella foenum-graecum* (fenugreek), *Cinnamomum verum* (cinnamon), and *Berberis aristata* (berberine source) may provide synergistic effects on glycemic control [37,38,39]. These multi-component formulations often target complementary mechanisms—enhancing insulin secretion, improving peripheral glucose uptake, modulating gut microbiota, and reducing postprandial glucose excursions [40]. For example, *Gymnema sylvestre* and bitter melon together have been reported to enhance β-cell regeneration and reduce intestinal glucose absorption more effectively than either alone [41]. Such strategies, if standardized and clinically validated, could be particularly useful for patients with inadequate response to single phytotherapeutics or for those seeking integrative approaches alongside standard pharmacotherapy.

Finally, the integration of phytotherapeutics into diabetes care reflects a growing interest in personalized and holistic approaches. Combining pharmacological agents with plant-derived compounds could offer synergistic effects, reduce pill burden by lowering the need for additional medications, and potentially improve patient adherence and satisfaction [42,43,44]. However, issues related to standardization of extracts, bioavailability, and long-term safety must be carefully addressed in future clinical trials [45,46].

Importantly, plant extracts cannot replace evidence-based pharmacological therapy. In our cohort, the most substantial improvements in glycemic, blood pressure, and body weight parameters were observed following dapagliflozin initiation, confirming the numerous pleiotropic effects of SGLT2-inhibitors [47,48]. In our study *Momordica charantia* did not demonstrate any significant effect on body weight although data from literature has documented certain anti-obesity effects [49] in rodents. Regarding blood pressure effects our results are similar with the ones from literature, where no statistically significant benefits were obtained in blood pressure reduction after *Momordica charantia* administration [50].

From a clinical perspective, these findings suggest that in patients who fail to achieve optimal glycemic control on dual antidiabetic therapy, adjunctive phytotherapeutic supplementation may be considered as an exploratory adjunct in selected patients, pending confirmation in randomized trials, particularly in real-world settings where polypharmacy and drug–drug interactions are concerns. This decision can have multiple benefits considering the danger of drug interaction especially in patients with numerous comorbidities. More than this, among diabetes mellitus Romanian patients the prevalence of plant supplements is high and they perceive this supplementation as improving the parameters of their disease, therefore the combination of plant supplement and allopathic drug could serve as an important tool for disease management [3,51].

The present study has several limitations. First, although a real-world control group was included, the study was observational and non-randomized, which may introduce residual confounding and limit causal inference. Second, the sample size was relatively modest (*n* = 155), and the duration of *Momordica charantia* supplementation was short (3 months), precluding conclusions regarding long-term efficacy and safety. Third, dietary intake, physical activity, and treatment adherence were not systematically monitored during follow-up; therefore, lifestyle changes may have contributed to, masked, or amplified the observed glycemic improvements. These limitations should be considered when interpreting the findings and underscore the need for larger, randomized, and longer-term studies.

Future studies should address these limitations by employing randomized, placebo-controlled designs with larger populations and longer follow-up durations. Mechanistic investigations are also required to clarify the pathways through which Momordica influences glucose metabolism, including its potential effects on insulin secretion, gut hormone regulation, and the gut microbiome. Furthermore, exploring the synergistic impact of Momordica with other emerging glucose-lowering agents could provide insights into integrated therapeutic strategies for type 2 diabetes.

## 5. Conclusions

In this study, adjunctive *Momordica charantia* supplementation, in patients receiving metformin and dapagliflozin therapy, was associated with additional improvements in glycemic control among patients with type 2 diabetes. The most notable benefits were observed in HbA1c and fasting plasma glucose, while changes in BMI, body weight, and blood pressure were modest. Comparative and multivariable analyses indicated that treatment allocation, rather than baseline comorbidity burden, was the primary determinant of glycemic improvement, highlighting the potential of therapeutic optimization to overcome baseline clinical complexity.

Our findings support the role of integrated pharmacological and phytotherapeutic strategies as pragmatic adjuncts to contemporary standard care in type 2 diabetes. However, given the observational design and non-randomized treatment allocation, larger, randomized, and longer-term studies are required to confirm the efficacy of *Momordica charantia*, clarify its mechanisms of action, and determine its contribution to sustained metabolic and cardiovascular outcomes.

## Figures and Tables

**Table 1:** Baseline demographic and clinical characteristics of the study population.

Parameter	Intervention (Momordica) *n* = 70	Control *n* = 85	*p*-Value
Age (years), mean ± SD	61 ± 9.0	60 ± 8.0	NS
Female sex, *n* (%)	38 (54.3)	45 (52.9)	NS
Diabetes duration ≥ 4 years, *n* (%)	32 (45.7)	37 (43.5)	NS
Peripheral neuropathy, *n* (%)	38 (54.3)	42 (49.4)	NS
Diabetic retinopathy, *n* (%)	14 (20.0)	16 (18.8)	NS
Chronic kidney disease, *n* (%)	13 (18.6)	14 (16.5)	NS
Hypertension, *n* (%)	36 (51.4)	42 (49.4)	NS
Heart failure, *n* (%)	13 (18.6)	15 (17.6)	NS

NS = not statistically significant.

**Table 2:** Evolution of anthropometric, hemodynamic, and glycemic parameters in the intervention group (*n* = 70).

Parameter	Unit	Baseline (T0) Mean ± SD	6 Months (T1) Mean ± SD	9 Months (T2) Mean ± SD
BMI	kg/m^2^	30.57 ± 2.21	29.56 ± 2.33	29.42 ± 2.41
Body weight	kg	95.90 ± 14.10	92.80 ± 13.90	92.50 ± 13.90
Systolic BP	mmHg	140.70 ± 12.60	133.50 ± 11.60	133.00 ± 9.70
Diastolic BP	mmHg	97.40 ± 15.90	93.50 ± 14.60	93.30 ± 13.50
HbA1c	%	7.82 ± 0.58	6.93 ± 0.30	6.34 ± 0.42
Fasting plasma glucose	mg/dL	138.40 ± 17.50	122.30 ± 13.10	113.30 ± 12.20

T0 = baseline (metformin monotherapy); T1 = 6 months after dapagliflozin add-on; T2 = 9 months from baseline after *Momordica charantia* supplementation.

**Table 3:** Paired within-group comparisons of anthropometric, hemodynamic, and glycemic parameters in the intervention group (*n* = 155).

Parameter	Comparison	Δ (Mean ± 95% CI)	*p* (Paired *t*-Test)	Cohen’s dz
BMI (kg/m^2^)	T0–T1	1.01 (0.85–1.17) ↓	<0.001	1.52
	T1–T2	−0.93 (−1.10–−0.76) ↓	<0.001	1.30
	T0–T2	0.08 (0.02–0.14)	0.006	0.34
Body weight (kg)	T0–T1	3.01 (2.38–3.64) ↓	<0.001	1.12
	T1–T2	0.31 (0.11–0.52)	0.003	0.37
	T0–T2	3.33 (2.69–3.96) ↓	<0.001	1.23
Systolic BP (mmHg)	T0–T1	7.19 (6.26–8.11) ↓	<0.001	1.82
	T1–T2	0.53 (−0.23–1.29)	0.177	0.16
	T0–T2	7.71 (6.38–9.05) ↓	<0.001	1.35
Diastolic BP (mmHg)	T0–T1	3.90 (3.28–4.52) ↓	<0.001	1.46
	T1–T2	0.19 (−0.49–0.86)	0.593	0.06
	T0–T2	4.09 (3.05–5.12) ↓	<0.001	0.93
HbA1c (%)	T0–T1	0.89 (0.79–0.99) ↓	<0.001	2.07
	T1–T2	0.59 (0.52–0.65) ↓	<0.001	2.00
	T0–T2	1.47 (1.36–1.58) ↓	<0.001	3.09
Fasting glucose (mg/dL)	T0–T1	16.1 (14.3–17.9) ↓	<0.001	2.11
	T1–T2	9.0 (7.7–10.4) ↓	<0.001	1.54
	T0–T2	25.1 (22.7–27.5) ↓	<0.001	2.44

Values are presented as mean paired differences (Δ) with 95% confidence intervals. ↓ indicates a significant decrease relative to the preceding time point. *p*-values are Bonferroni-corrected (α_adj = 0.0167). Cohen’s dz ≥ 0.8 denotes a large effect size.

**Table 4:** Multivariable linear regression models assessing predictors of HbA1c change between T1 and T2 (ΔHbA1c T1–T2).

Predictors Included	Model F (*df*)	ANOVA *p*-Value	Key Predictors (β, *p*)
Group (intervention vs. control)	—	<0.001	Group (β ≈ −0.38, *p* < 0.001)
Group, HF	1.59	0.21	Group (*p* < 0.001); HF (β ≈ 0.15, *p* = 0.21)
Group, HF, HTN	2.47	0.09	Group (*p* < 0.001); HF (β ≈ 0.26, *p* ≈ 0.05); HTN (β ≈ −0.24, *p* ≈ 0.07)
Group, HF, HTN, CKD	1.87	0.14	Group (*p* < 0.001); others NS
Group + comorbidities + DD	1.12	0.36	Group (*p* < 0.001); all others NS
Group, age, sex, HbA1c T0, HbA1c T1	0.33	0.72	Group (*p* < 0.001); others NS

HF = heart failure; HTN = hypertension; CKD = chronic kidney disease; DD = diabetes duration; NS = not significant.

## Data Availability

The original contributions presented in this study are included in this article. Further inquiries can be directed to the first authors.

## Abbreviations

The following abbreviations are used in this manuscript:

AGE	advanced glycation end products
BMI	body mass index
CKD	chronic kidney disease
DBP	diastolic blood pressure
GLP-1	glucagon-like peptide 1
GLUT-4	glucose transporter type 4
HbA1c	glycated hemoglobin
HF	heart failure
HTN	hypertension
PNP	peripheral neuropathy
PPAR	peroxisome proliferator-activated receptor
RP	retinopathy
SBP	systolic blood pressure
SGLT2	sodium-glucose cotransporter 2
T2DM	type 2 diabetes mellitus
